# Environmental Influence on Bacterial Lipid Composition: Insights from Pathogenic and Probiotic Strains

**DOI:** 10.1021/acsomega.4c03778

**Published:** 2024-08-29

**Authors:** Justyna Walczak-Skierska, Agnieszka Ludwiczak, Ewelina Sibińska, Paweł Pomastowski

**Affiliations:** † Centre for Modern Interdisciplinary Technologies, Nicolaus Copernicus University in Toruń, Wileńska 4 Str., Toruń 87-100, Poland; ‡ Faculty of Biological and Veterinary Sciences, Nicolaus Copernicus University in Toruń, Lwowska 1 Str., Toruń 87-100, Poland

## Abstract

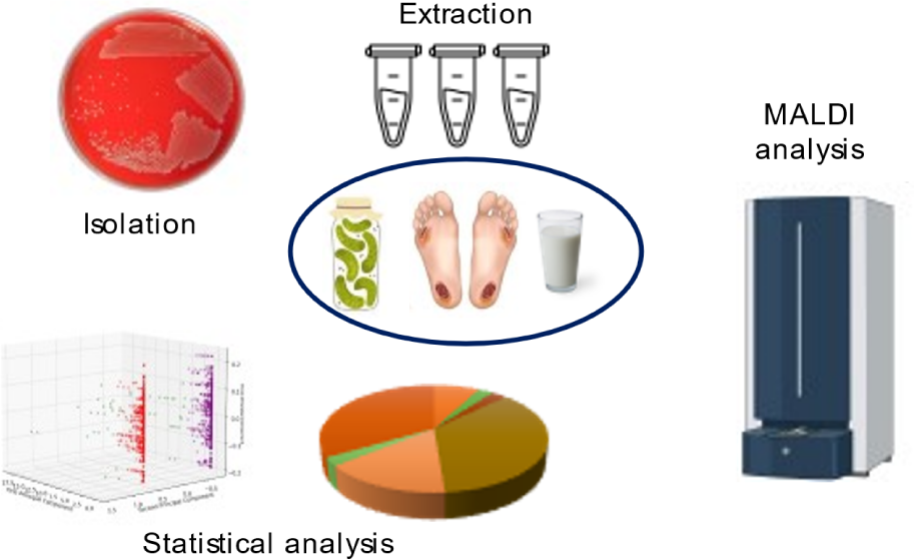

The lipid composition of bacterial membranes is pivotal in regulating bacterial physiology, pathogenicity, and interactions with hosts. This study presents a comprehensive analysis of bacterial membrane lipid profiles across diverse Gram-positive and Gram-negative species. Utilizing matrix-assisted laser desorption/ionization (MALDI) in conjunction with advanced chemometric tools, we investigate the influence of environmental factors, isolation sources, and host metabolism on bacterial lipid profiles. Our findings unveil significant variations in lipid composition attributed to factors such as carbon/energy availability and exposure to chemicals, including antibiotics. Moreover, we identify distinct lipidomic signatures associated with pathogenic and probiotic bacterial strains, shedding light on their functional properties and metabolic pathways. Notably, bacterial strains isolated from clinical samples exhibit unique lipid profiles influenced by host metabolic dysregulation, particularly evident in conditions such as diabetic foot infections. These results deepen our understanding of the intricate mechanisms governing bacterial membrane lipid biology and hold promise for informing the development of innovative therapeutic and biotechnological strategies.

## Introduction

The human gastrointestinal tract represents a dynamic ecosystem characterized by a remarkable diversity of microbial organisms, surpassing a remarkable census of 100 trillion. This diverse consortium collectively comprises the intestinal microflora, a complex assemblage that plays an active role in modulating human health.^1^ Within this intricate microbial milieu, bacterial populations are stratified based on their physiological effects on the host, delineating between those that confer beneficial outcomes and those that elicit deleterious responses.^2^

Among the cohort of beneficial microorganisms, probiotics have risen to prominence, garnering considerable scientific interest owing to their potential therapeutic effects on human health.^3^ Coined by Lilly et al. in 1965, the term “probiotics” denotes living microorganisms that, when administered in adequate quantities, confer beneficial health outcomes to the host.^4^ While historically associated predominantly with genera such as *Lactobacillus* and *Bifidobacterium*, the probiotic landscape has expanded to encompass a broader array of bacterial strains, including *Lactococcus*, *Enterococcus*, *Bacillus*, and *Pseudomonas* which are currently under investigation for their putative probiotic attributes.^5−7^ Probiotics exhibit a diverse array of functional characteristics, spanning from the promotion of digestive processes and modulation of cholesterol levels to the mitigation of obesity and modulation of immune responses. Certain strains have demonstrated the capacity to synthesize short-chain fatty acids (SCFAs), modulate gut microbiota composition, and manifest antiobesity and cholesterol-reducing effects.^7−10^ Moreover, probiotics are capable of synthesizing antimicrobial compounds such as bacteriocins and peptides, thereby augmenting the host’s innate defense mechanisms.^7−10^ Beyond their digestive functions, probiotics exert broader influences on host physiology, including modulation of immune responses, exertion of anticancer effects, and modulation of mood and behavior via interactions with the central nervous system.^7,11^

In stark contrast, pathogenic microorganisms have evolved intricate mechanisms to subvert host defenses and exploit host cellular machinery for their survival and proliferation. These pathogens, spanning viruses, bacteria, fungi, and parasites, engage in complex interactions with host cells, often manipulating host cell lipids to their advantages.^12^ Lipids, particularly those within the hydrophobic metabolome or lipidome, serve as attractive targets for pathogens to modulate host cell processes. By manipulating lipid metabolism, pathogens enhance invasion, replication, and evasion of host immune responses, thereby ensuring their survival and propagation.^13,14^ Lipids, besides serving as nutrient sources, play pivotal roles in modulating host cell metabolism, physiology, and membrane trafficking. Pathogens skillfully target host cell lipids such as cholesterol, triacylglycerols (TAGs), and phosphoinositides (PIs) to facilitate intracellular survival and manipulate host cell functions.^15^

Furthermore, bacterial cells possess sophisticated molecular machinery that enables them to dynamically respond to changes in membrane structure. This intricate regulatory control is essential for coordinating the synthesis of novel lipids and the modification of existing ones, facilitating the remodeling of membrane structures. These adaptive responses are vital for bacterial survival and adaptation to various extrinsic stressors, including fluctuations in temperature, hydration levels, and nutrient availability.^16,17^ Bacterial membranes constitute a complex assembly of lipids, predominantly consisting of glycerophospholipids, glycerolipids, and prenol lipids. Among these lipid classes, glycerophospholipids predominate as the primary constituents.^18^ Nonetheless, the lipid composition of bacterial membranes exhibits significant variability among different bacterial species, reflecting their diverse physiological and ecological niches. Notably, Gram-negative and Gram-positive bacteria are distinguished by distinct phospholipid compositions. For example, phosphatidylethanolamine (PE) emerges as a characteristic phospholipid abundant in Gram-negative bacteria, where it plays essential roles in maintaining membrane integrity and cellular physiology. Conversely, certain Gram-positive bacteria, such as *staphylococci* and *streptococci*, lack PE in their membrane lipid repertoire. These disparities underscore the intricate relationship between lipid composition and bacterial physiology, illustrating the diverse adaptive strategies employed by bacteria to thrive in their respective environmental habitats.^18−20^

The main aim of this study is to thoroughly profile the lipidomic compounds of various bacterial groups, particularly focusing on Gram-positive (G (+)) and Gram-negative (G (−)) species. Furthermore, the research endeavors to undertake a comparative analysis of lipid profiles between pathogenic and probiotic bacterial strains, aiming to discern distinctive lipidomic signatures associated with their respective functional roles. Additionally, the investigation seeks to unravel variations in lipid composition attributable to diverse isolation sources, spanning from conventional substrates like milk and cheese to unconventional sources such as beetroot and clinical samples sourced from patients. To achieve these objectives, analytical techniques, including matrix-assisted laser desorption/ionization (MALDI) paired with chemometric tools, were employed. By using these methodologies, this study aims to elucidate the complex and multifaceted roles of bacterial lipids in health and disease, thereby enhancing our understanding of microbial lipid biology.

## Materials and Methods

### Chemicals

Methanol, acetonitrile, trifluoroacetic acid (TFA), chloroform, sodium chloride, and water of high purity grade were procured from Sigma-Aldrich (Steinheim, Germany). The matrix used, 2,5-dihydroxybenzoic acid (DHB), and the mass standards kit for calibration were also sourced from Sigma-Aldrich.

### Preparation of Samples

For the analysis, 24 bacterial isolates representing 10 different species were selected: *Lactococcus lactis*, and *Lactobacillus plantarum* (which are probiotics bacteria), *Staphylococcus aureus*, *Staphylococcus epidermidis*, *Enterococcus faecalis*, *Pseudomonas aeruginosa*, *Escherichia coli*, *Proteus mirabilis*, *Klebsiella pneumoniae*, and *Citrobacter freundii* (which are pathogenic bacteria). The selected strains were isolated from various sources; clinical: diabetic foot infection (marked as DFI),^21^ urine from patient with prostate cancer (U),^22^ pressure ulcers (PU) and environmental: silage^23,24^ and dairy products. All strains were inoculated on Tryptic Soy Agar (Sigma-Aldrich, Steinheim, Germany) medium and incubated at 37 °C for 18 h.

### Lipid Extraction from Bacteria

The extraction of the lipid fraction from bacteria followed the procedure outlined by Folch et al. with some modifications.^25^ Bacterial pellets, approximately 50 mg for each analyzed strain, were dissolved in a 2 mL mixture of chloroform/methanol (2:1, vol/vol) and 0.5 mL of sodium chloride (0.05 M NaCl). The solutions underwent ultrasonication for 10 min at room temperature, followed by shaking for 10 min on a rotary shaker (200 rpm), and subsequent centrifugation at 5000 rpm for 20 min. After centrifugation, the lower layer was collected, and the process was repeated by adding 0.5 mL of chloroform to the upper phase. The lower phases were combined and evaporated using a Labconco CentriVap DNA concentrator (Kansas City, USA). The extracted lipids were stored at −20 °C for future analysis.

### MALDI-TOF MS Analysis

Mass spectrometric measurements were conducted using a MALDI-TOF/TOF MS instrument (Bruker Daltonics, Bremen, Germany). The instrument, equipped with a modified neodymium-doped yttrium aluminum garnet (Nd: YAG) laser (1-kHz Smartbeam-II, Bruker Daltonik) operating at a wavelength of 355 nm, was employed for all measurements. The extraction voltage was set at 25 kV, and gated matrix suppression was applied to prevent detector saturation by matrix ions. All spectra were acquired in reflector positive mode within a *m*/*z* range of 200–1400 at 80% laser power and a global attenuator of 50%. FlexControl and flexAnalysis software (both from Bruker Daltonik) were used for acquisition and processing of all mass spectra, respectively.

The extracted lipids were dissolved in 20 μL of methanol. One μL of extracts was placed on a MTP 384 target plate ground steel (Bruker Daltonics, Bremen, Germany) and covered with 1 μL of α-cyano-4-hydroxycinnamic acid matrix solution. The matrix was prepared as a saturated solution in TA30 (acetonitrile and 0.1% trifluoroacetic acid in water 30:70 (v/v)). The mass spectra were calibrated by using the cesium triiodide cluster. The calibrant was prepared by dissolving 5 mg of CsI in 0.5 mL of methanol and adding 0.5 mL of a 20 mg/mL solution of 2,5-dihydroxybenzoic acid in methanol (anchor diameter 800 μm; Bruker Daltonik GmbH, Bremen, Germany).

Identification of all lipid species was achieved by utilizing the LIPID MAPS online database (http://www.lipidmaps.org).

### Statistical Analysis

Several tools from the Python ecosystem and PS IMAGO PRO 9.0 package (IBM SPSS Statistics) were used to conduct the analyses. Data transformation and aggregation processes were conducted using the Pandas library (McKinney, 2010) for the Python programming language. K-Means clustering and Principal Component Analysis (PCA), were implemented using the Scikit-learn library. Data visualizations were created using Matplotlib. Fisher’s Exact Test and the Mann–Whitney U test were utilized to assess the differences between categorical variables and two independent samples, respectively. The significance levels were predetermined, with a p-value threshold of 0.05 indicating statistical significance, denoted on the graphs by a single asterisk (*). Further, a higher significance level with a p-value threshold of 0.01 was also used, which was represented on the charts by two asterisks (**). The Fisher’s z-test was performed to compare the correlation coefficients between the pathogen and probiotic correlation coefficients.

Clustering of all observations utilizing the K-means algorithm was conducted following the reduction of data dimensionality to 2 or 3 principal components, facilitating the visualization of data distribution across the plane of the 3 principal components and segmentation into groups G+ and G- distinguished by the average signal intensity. In addition, Cluster analysis utilizing the K-means algorithm enabled the differentiation of the data into two clusters of pathogenic and probiotic bacteria. The data were normalized to prevent any attribute from dominating the clustering process due to the scale of values. Dimensionality reduction was applied using the Principal Component Analysis (PCA) method to transform data into a two-dimensional space. The results are presented in a scatter plot, where colors correspond to the membership in respective clusters.

## Results and Discussions

### Diversity Across Gram-Positive and Gram-Negative Bacterial Groups

**Figure 1:**
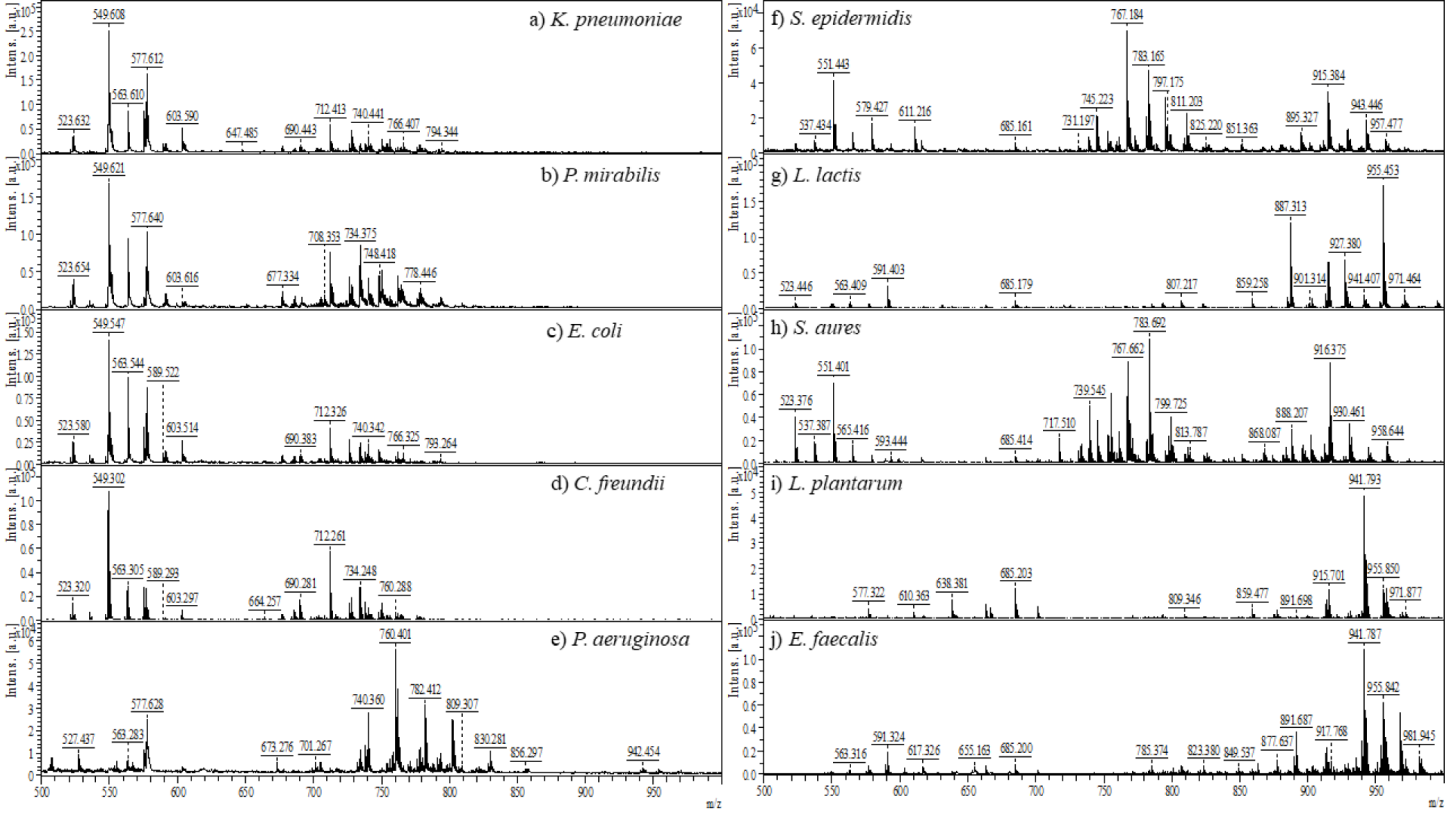
Mass spectra obtained via MALDI in negative ionization mode for Gram-negative bacteria: a) *K. pneumoniae*, b) *P. mirabilis*, c) *E. coli*, d) *C. freundii*, e) *P. aeruginosa*, and Gram positive bacteria: f) *S. epidermidis*, g) *L. lactis*, h) *S. aures*, (i) *L. plantarum*, j) *E. faecalis*.

Bacterial species investigated in this study included a diverse range of ten strains, comprising five Gram-negative and five Gram-positive bacteria (Table 1). These strains were sourced from various environments such as diabetic foot infection, pressure uclers, urine specimens from prostate cancer patients, fermented food products, and dairy items. Subsequent analysis involved subjecting these isolates to MALDI-TOF MS examination, spanning a mass scanning range from *m*/*z* 200 to 1400. Notably, the desorption and detection of ions derived from large and polar biomolecules, including ribosomal proteins, peptides, and carbohydrates within the bacterial colonies, were not observed. Figure 1 illustrates the MALDI-TOF MS mass spectra acquired in positive ion mode from colonies of the ten bacterial species under investigation. Predominantly, intact lipids within the *m*/*z* 700–900 mass range were the major ions detected, highlighting the lipidomic profile of these bacterial isolates. Additionally, lipid ion signals observed in Gram-negative bacterial colonies (Figure 1a–e) exhibited notably higher intensities compared to those in Gram-positive bacteria (Figure 1f–j). Several factors contribute to the detection of reduced ion signals from Gram-positive bacterial colonies by MALDI-TOF MS. First, Gram-positive bacteria envelope differs significantly from that of Gram-negative bacteria, primarily due to the absence of an outer membrane (OM). The OM in Gram-negative bacteria provides a protective barrier, contributing to membrane stability and structural integrity. In contrast, Gram-positive bacteria have a much thicker peptidoglycan layer with teichoic acids and surface proteins. Furthermore, absence of the OM in Gram-positive bacteria means they lack the lipid bilayer that facilitates the desorption process in MALDI-TOF MS. The glycolipid (LPS) composition of the OM in Gram-negative bacteria enhances ionization, leading to stronger ion signals. Conversely, the rigid and complex peptidoglycan structure in Gram-positive bacteria can result in reduced ion signals.^26^ This peptidoglycan layer, comprising a polymer of sugars and amino acids, forms a robust structural barrier that complicates the release of lipids from the inner cell membrane of Gram-positive bacteria.^27^ Consequently, the ion signals derived from Gram-positive bacterial colonies, exemplified by *Enterococcus faecalis*, exhibit complex spectra with relatively low signal intensity, as depicted in Figure 1j. Additionally, MALDI mass spectra of both Gram-negative and Gram-positive bacterial colonies were also recorded in negative ion mode using MALDI. However, this analytical approach did not yield sufficient lipid ion signals to discriminate between different bacterial species, highlighting the inherent challenges in lipidomic analysis using MALDI-TOF MS.

**Table 1:** Bacterial Species Examined in This Investigation

gram positive	gram negative
*Lactobacillus plantarum*	*Citrobacter freundii*
*Enterococcus faecalis*	*Pseudomonas aeruginosa*
*Staphylococcus aures*	*Escherichia coli*
*Staphylococcus epidermidis*	*Proteus mirabilis*
*Lactococcus lactis*	*Klebsiella pneumoniae*

**Figure 2:**
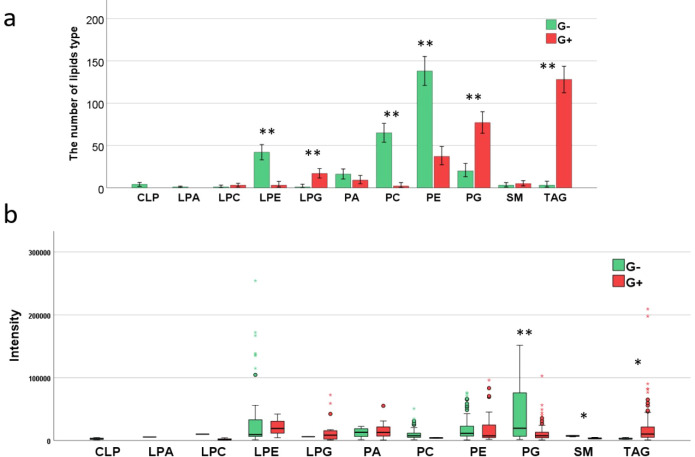
Number of lipids type in Gram negative and Gram positive with the number of lipids in the lipids types, defined as N (a) and intensity of the identified class of lipids (b). Statistical significance was assessed using Fisher’s Exact Test (a) and the Mann–Whitney U test (b), with p-values indicated by red asterisks: *p < 0.05, **p < 0.01.

Moreover, the MALDI-TOF MS analysis facilitated the differentiation between Gram positive (*N* = 281) and Gram negative (*N* = 294) bacteria. The lipid composition varied significantly between these groups, as illustrated in Figure 2a. For G- bacteria, phosphatidylethanolamine was the most prevalent lipid type, accounting for 46.9% of the lipidome, while triacylglycerols were predominant in G+ bacteria, comprising 45.6% of their lipid profile. Additionally, Gram negative bacteria exhibited a significantly higher presence of lyso-phosphatidylethanolamine (LPE) compared to Gram positive bacteria. Conversely, Gram positive bacteria demonstrated a higher quantity of phosphatidylglycerol (PG) and TAG lipids. Notably, the Cardiolipin (CLP) and lyso-phosphatidic acid (LPA) lipid classes were absent in Gram positive bacteria (Figure 2a).

Differences in the intensity of lipid signals between G (−) and G (+) were observed (Figure 2b). The higher signal intensities for LPE and TAG lipids were detected in G (+), whereas phosphatidylcholine (PC), PG, and sphingomyelin (SM) exhibited higher intensities in Gram negative. However, the differences in signal intensity for lyso-phosphatidylglycerol (*p* = 0.667), phosphatidylcholine (*p* = 0.232), phosphatidylethanolamine (*p* = 0.306), and phosphatidic acid (*p* = 0.932) were not statistically significant between the G (+) and G (−) groups.

**Figure 3:**
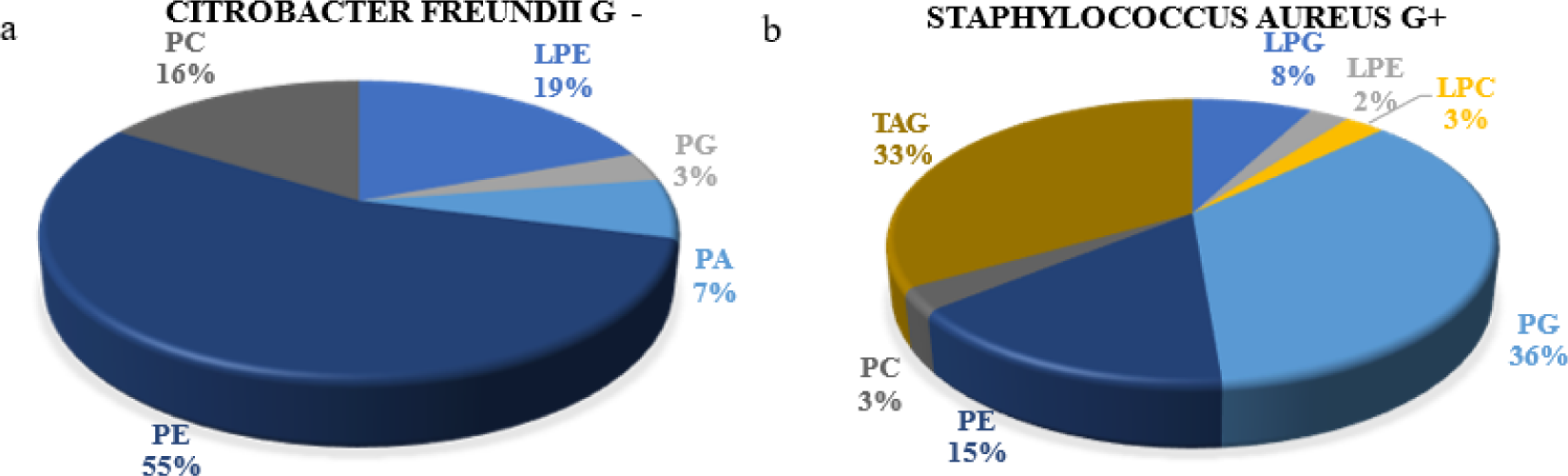
Lipid species in a) *Citrobacter freundii* and b) *Staphylococcus aureus*.

Our investigation (Supplementary S1) unveiled considerable qualitative disparities in lipid compositions between Gram-positive and Gram-negative bacterial species. A case in point involves the analysis of *Citrobacter freundii* (−) and *Staphylococcus aureus* (+) strains isolated from a patient with diabetic foot infection, where notable distinctions in the cytoplasmic membrane lipid makeup were observed in Figure 3. In *Citrobacter freundii*, phosphatidylethanolamine dominates the lipid profile, constituting over half (17 molecules of lipids, which constituted 55% of the total lipid population) of the total lipids, accompanied by lower proportions of lyso-phosphatidylethanolamine (6 LPE, 19%), phosphatidylcholine (5 PC, 16%), phosphatidic acid (2 PA, 7%), and phosphatidylglycerol (1 PG, 3%) (Figure 3a). Conversely, *S. aureus* exhibits a prevalence of anionic phosphatidylglycerol (14 PG, 36%) and triacylglycerols (13 TAG, 33%), complemented by phosphatidylethanolamine (6 PE, 15%), lyso-phosphatidylglycerol (3 LPG, 8%), phosphatidylcholine (1 PC), lyso-phosphatidylcholine (1 LPC, each 3%), and lyso-phosphatidylethanolamine (1 LPE, 2%) (Figure 3b). Gram-positive bacteria feature a thick cell wall resulting from the fusion of murein with other polysaccharide polymers, such as teichoic acid or glycerol polymer. Murein can also bind to existing proteins, forming multilayered structures. On the other hand, Gram-negative bacteria possess an additional outer membrane layer, consisting of proteins and lipopolysaccharides (LPS). Lipid A contains a sugar core composed of two glucosamine molecules. In the structure of this bacteria, the inner part mainly consists of glycerophospholipids, while the outer part is rich in highly negatively charged lipopolysaccharides.^26,28^.

In *C. freundii*, the fatty acid content ranged from C10 to C20 with a degree of unsaturation between 1 and 4. The unsaturation content for this bacteria accounted for 71% of all identified lipid molecules. For all identified lipid molecules, the acyl tail contained between 10 to 24 carbon atoms, with a degree of unsaturation ranging from 0 to 6. A total of 132 fatty acid combinations in lipid molecules were identified (Table S1). The conducted studies revealed a significant diversity of membrane lipids, dependent on factors such as the wide variety of chain lengths, branching, headgroup types, degree of unsaturation, formation of biochemical rings, as well as modifications with sugar residues and other functional groups of different biosynthetic origins.

The complexity of acyl chains forming membrane lipids encompasses a diverse array of structural variations and compositional intricacies, directly influencing the multifaceted nature of membrane properties. This intricacy emerges from substantial diversity in acyl chain length, modifications regarding the number, position, and cis–trans stereo isomers of double bonds, as well as the presence of methyl branching.^29^ Adaptations in response to varying growth conditions and environmental stimuli facilitate adjustments within the physical and structural characteristics of the membrane, ensuring optimal functionality.^30^ Gram-negative bacteria predominantly feature acyl chains with carbon atom counts ranging from 16 to 18 in their membrane lipids, while Gram-positive bacteria are characterized by the prevalence of fatty acids such as C16, C18, and C20 (Table S1). Notably, acyl chain length emerges as a pivotal factor governing membrane thickness modulation. Furthermore, it has been observed that Gram-negative bacteria tend to synthesize a higher proportion of both saturated and unsaturated fatty acids with even carbon numbers, whereas odd-numbered fatty acids are predominantly found in Gram-positive bacteria. Across both Gram-positive and Gram-negative bacterial species, a significant proportion of identified fatty acids consist of polyunsaturated fatty acids (PUFA), exceeding 40% in most cases. *Lactobacillus plantarum* (beetroof) stands out with PUFA comprising over 83% of its identified fatty acids, while *Proteus mirabilis* (urine) exhibits a lower proportion, approximately 41%. Monounsaturated fatty acids (MUFA) range from 11% to 25% for Gram-negative bacteria and from 5% to 29% for Gram-positive bacteria, highlighting their variability across bacterial groups. In contrast, saturated fatty acids (SFA) constitute the smallest percentage among all identified fatty acids (Table S1).

**Figure 4:**
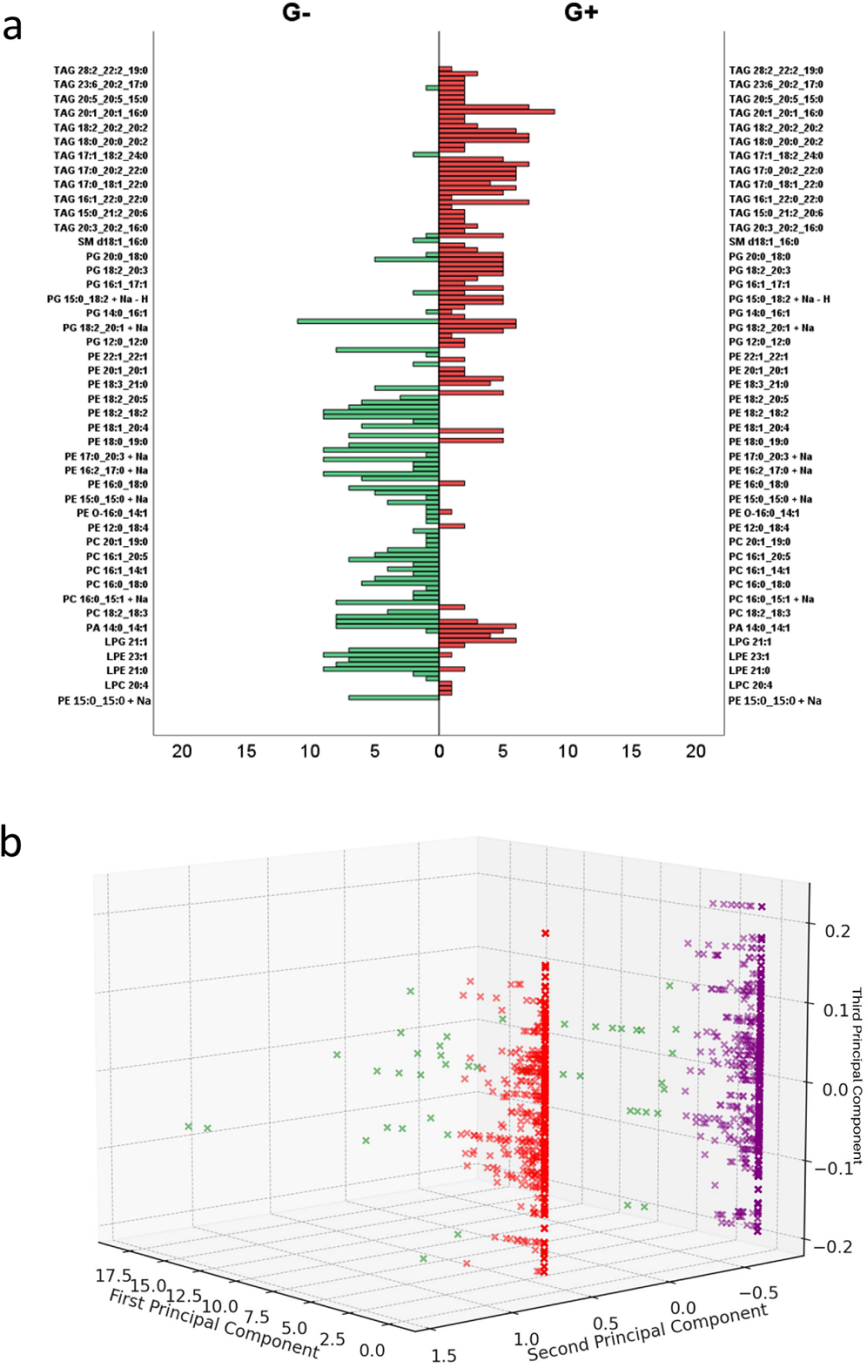
Detailed list of the identified lipids in G (+) and G (−) with the number of identified lipids (a). The results of the K-means clustering present the data distribution across the plane of the three principal components. Each point represents an observation, with its color indicating membership in one of the clusters (b).

The observed lipid profiles demonstrated varied distributions for G (+) and G (−) bacteria (Figure 4). Within the most represented lipid group of triacylglycerols in Gram positive, a high number of specific TAG types were identified, including TAG 20:1_20:1_16:0, TAG 16:0_22:0_22:2, TAG 17:0_20:2_22:1, TAG 18:0_20:0_20:2, TAG 18:0_20:1_20:2, and TAG 20:1_20:2_16:0, as well as phosphatidylglycerol species like PG 18:2_20:1 + Na and PG 10:0_10:0, which were more prevalent compared to the Gram negative group. Conversely, in the G- group, dominant lipid classes included phosphatidylethanolamine with PE 16:1_17:0 + Na, PE 16:2_18:2 + Na, PE 18:0_18:0, PE 18:2_16:2, PE 18:2_18:2, phosphatidylcholine with PC 16:1_18:3, PC 16:0_14:0, PC 16:1_18:2, and lyso-phosphatidylethanolamine with LPE 21:0, LPE 21:1, and LPE 23:1 (Figure 4a).

The PCA analysis revealed that the three principal components together account for approximately 70% of the variance (Figure 4b). Visualization of the first three principal components of data using the K-means clustering algorithm demonstrated differences in the average signal intensity for various lipids in each cluster, facilitating the classification of bacteria into G (+) and G (−). Cluster 1 exclusively contains G- bacteria (*N* = 1470) with an average signal intensity of 2184, while Cluster 2 contains only G+ bacteria (*N* = 1613 observations) with an average signal intensity of 1909. Cluster 3 is assigned a small number of both G (+) and G (−) bacteria (*N* = 19 and 26, respectively).

Extensive research has demonstrated that fatty acids possessing two or more double bonds, starting from C 18:2, exhibit heightened activities, including antibacterial properties, surpassing those of saturated fatty acids. While saturated fatty acids demonstrate efficacy against microorganisms with shorter chain lengths, monounsaturated and polyunsaturated fatty acids, characterized by longer chains, display enhanced effectiveness. The precise positioning of double bonds emerges as a critical determinant in the functional capabilities of long-chain fatty acids within bacterial membranes.^31,32^

### Contrasting Lipidomic Signatures of Pathogenic versus Probiotic Bacterial Strains

Our research also focused on comparing the lipid profiles of pathogens and probiotics. In the context of microorganisms, lipid composition can significantly influence their pathogenic or probiotic properties. This study aimed to investigate characteristic differences in lipid composition between pathogenic and probiotic bacterial strains, which could have important implications for understanding their mechanisms of action and for the development of new therapeutic and prophylactic strategies. By introducing these differences in lipid profiles, we aim to enhance the understanding of the role of lipids in microorganism-host interactions.

The probiotic bacteria included in our analysis are strains such as *Lactococcus lactis*, and *Lactobacillus plantarum*. The pathogenic bacteria analyzed are strains such as *Staphylococcus aureus*, *Staphylococcus epidermidis*, *Enterococcus faecalis*, *Pseudomonas aeruginosa*, *Escherichia coli*, *Proteus mirabilis*, *Klebsiella pneumoniae*, and *Citrobacter freundii*. As a result of our conducted research, we successfully identified specific lipid molecules, including LPG 12:0, LCP 18:1, LPC 20:4, PG 12:0_12:0, PE 16:0_18:0, and PG 16:0_20:0, within two probiotic strains, namely *L. plantarum* and *L. lactis*. Remarkably, these lipid signatures were absent in the analyzed pathogenic strains. Furthermore, the probiotic strains were isolated from fermented foods and dairy products, suggesting their potential role in food fermentation processes and probiotic formulations. The presence of these characteristic lipid signals exclusively in the probiotic strains isolated from environmental sources implies their potential significance as biomarkers or discriminative compounds compared to other bacteria typically categorized as pathogens. This finding underscores the importance of lipid profiling in discerning between probiotic and pathogenic strains, offering insights into their distinct metabolic pathways and functional properties.

**Figure 5:**
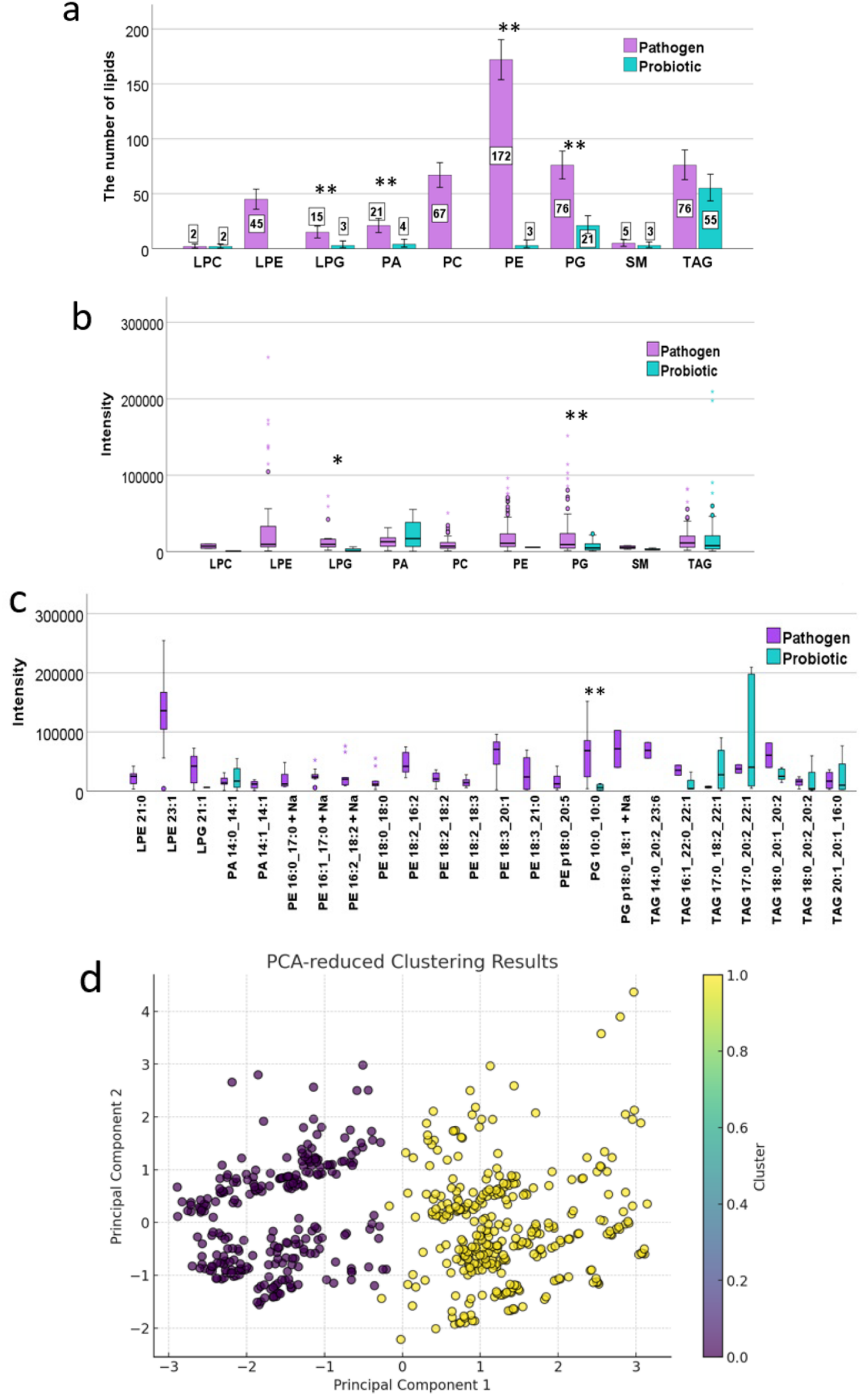
Analysis of lipid number (a) and intensity of the types of lipids signal differences (b,c) between probiotic and pathogenic bacteria. The data were presented after dimensionality reduction using PCA revealing distinct patterns (d). Cluster 0 (purple) and Cluster 1 (yellow) are differentiated by two colors in the visual representation. Fisher’s Exact Test (a) and the Mann–Whitney U test (b,c), with p-values indicated by red asterisks: *p < 0.05, **p < 0.01.

Distinct numbers and types of lipids were identified between pathogenic and probiotic bacteria (Figure 5). LPE and PC were presented only in pathogen samples. A statistically significant greater number of PE and PG lipids were observed in pathogenic bacteria compared to probiotic bacteria (Figure 5a). Furthermore, the intensity of lipid signals varied between pathogenic and probiotic bacteria. Notably, a statistically significant higher intensity for LPG and PG was found in pathogenic bacteria than in probiotic bacteria (Figure 5b).

We also identified differences in the intensity of specific types of lipids. Higher signal intensities were observed for LPG 21:1, triacylglycerols: TAG 18:0_20:1_20:2, and TAG 18:0_20:2_20:2 in pathogen samples. Certain signals were exclusively present in pathogen samples, including LPE 23:1, PA 14:1_14:1, phosphatidylethanolamines: PE 16:0_17:0+Na, PE 16:2_18:2+Na, PE 18:2_16:2, PE 18:2_18:2, PE 18:3_20:1, PE 18:3_21:0, and TAG 14:0_20:2_23:6 (Figure 5c). The higher number of lipid types in pathogenic bacteria compared to probiotic bacteria can be attributed to their differing roles and environments. Pathogens need complex and diverse lipids to maintain structural integrity and functionality under infection-related stresses, such as oxidative stress and antimicrobial pressures. They exhibit high metabolic flexibility, requiring a broader array of lipids for adaptability and various metabolic pathways. Diverse lipids also help pathogens modify surface structures to evade the host immune system. In contrast, probiotic bacteria, residing in stable and nutrient-rich environments like the gut, have a more streamlined lipid composition to support gut health and nutrient absorption without extensive defensive mechanisms.^15,33,34^

Data reduction to principal components via PCA facilitated the segregation into two distinct clusters. Probiotic species, such as *L. plantarum* and *L. lactis*, were predominantly found in cluster 0, characterized by a lower average signal intensity (16,624.30) than cluster 1, with TAG 20:1_20:1_16:0 being the dominant lipid type. Cluster 1 had a higher average signal intensity (19,778.26), with PA 14:0_14:1 as the dominant lipid type (Figure 5d).

In order to compare pathogenic and probiotic bacteria, lipid saturation levels were analyzed across different bacterial species. The study included a diverse range of bacterial strains, encompassing both pathogenic and probiotic species. Lipid saturation levels were quantified and compared using statistical methods to evaluate potential differences between the two groups.

**Figure 6:**
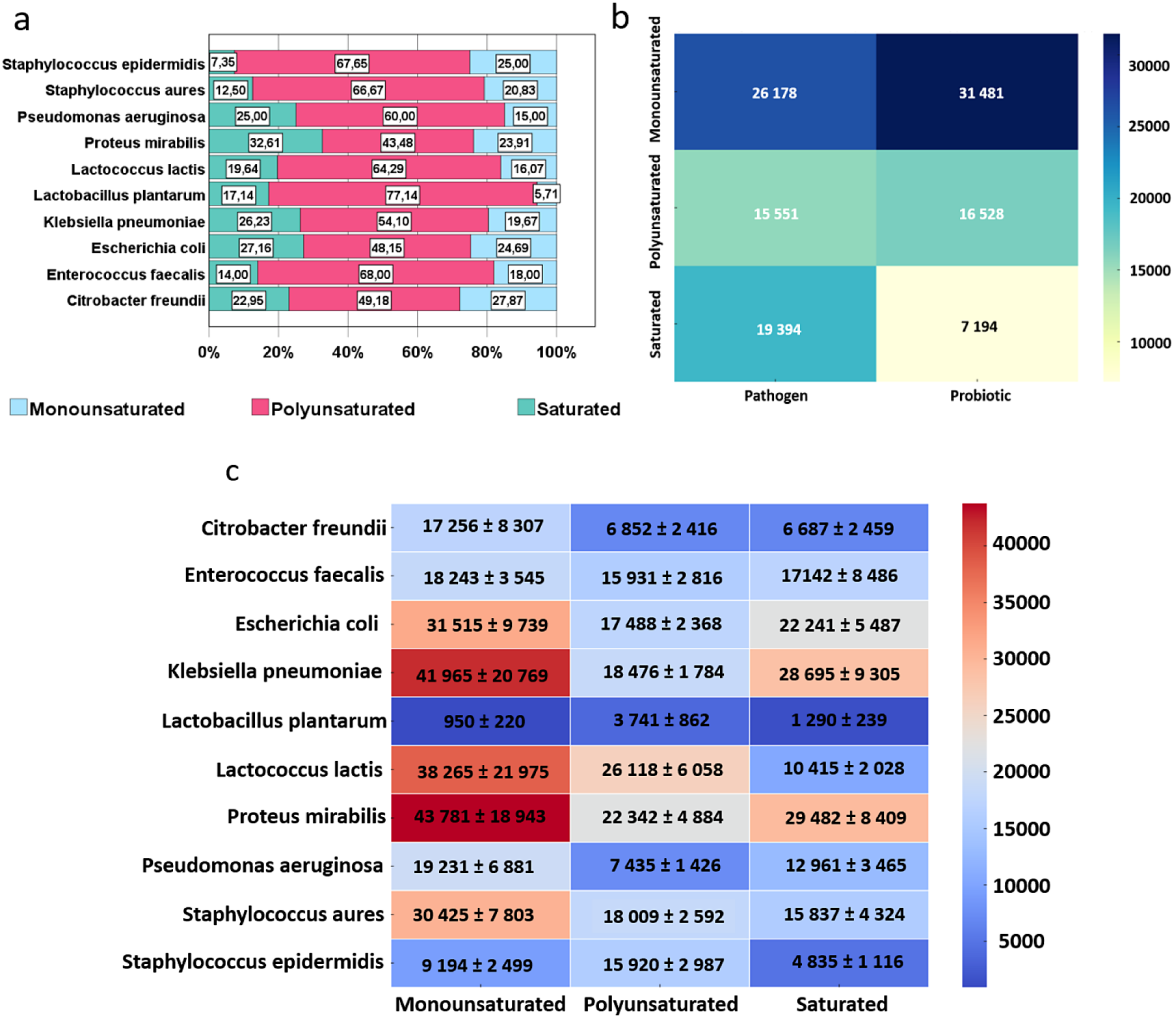
Lipid saturation profiles across analyzed bacterial species. a) details of the lipid saturation profiles specific to the bacterial species, b) the heatmap with the mean intensity values for three normalized types of lipid saturation (saturated, monounsaturated, and polyunsaturated) across pathogenic and probiotic bacteria, c) the heatmap illustrated the average intensity values for each combination of bacterial species and lipid saturation with the standard error (SE) calculated based on intensity.

The levels of lipid saturation varied across bacterial species (Figure 6). Among all bacterial species, polyunsaturated fatty acid in molecules of lipids were dominant, with a significant prevalence in *S. aureus* and *S. epidermidis*. Monounsaturated fatty acid were more prevalent than saturated ones in *C. freundii*, *S. aureus*, and *S. epidermidis*. For *E. coli* and *L. lactis*, a similar quantity of monounsaturated fats was obtained (Figure 6a). Additionally, the average intensity of lipids varied between pathogenic and probiotic bacteria. The Mann–Whitney test revealed statistically significant differences in signal intensity for different types of saturation depending on the type of bacteria (pathogenic, probiotic). A statistically significant difference in signal intensity for polyunsaturated and saturated fatty acids between probiotic and pathogenic bacteria was demonstrated (*p*= 0.0103, *p*= 0.00399, respectively) (Figure 6b). The average intensity varies for different lipid types across various species (Figure 6c). The highest intensities were recorded for monounsaturated fatty acids in *P. mirabilis*, *K. pneumoniae*, and *L. lactis*. Conversely, the highest intensity for saturated fatty acids was observed in *P. mirabilis* and *K. pneumoniae*. For *E. faecalis*, similar signal intensity values were demonstrated regardless of the saturation degree of fatty acids.

The lipid profile of probiotic and pathogenic bacteria displays significant diversity. Probiotics compete with pathogens for binding sites and nutrients. Furthermore, probiotic bacteria effectively block pathogen colonization through adhesion.^35^ In Kishino et al.’s study,^36^*L. plantarum* was found to produce enzymes involved in PUFA saturation, resulting in various intermediates observed at higher levels in microbiota-colonized mice compared to germ-free mice. Linoleic acid Δ9 hydratase, identified in *L. plantarum*, initiates PUFA saturation, with similar activity found in *Lactobacillus acidophilus*. PUFAs serve as selective substrates for host microorganisms, providing energy for gut microbiota and activating specific metabolic pathways. Notably, gut bacteria and their metabolites mutually interact with the host. Regarding the health benefits of PUFAs as prebiotics, studies suggest omega-3 PUFAs offer the greatest benefits, followed by MUFAs, omega-6 PUFAs, and SFAs.^37^

**Figure 7:**
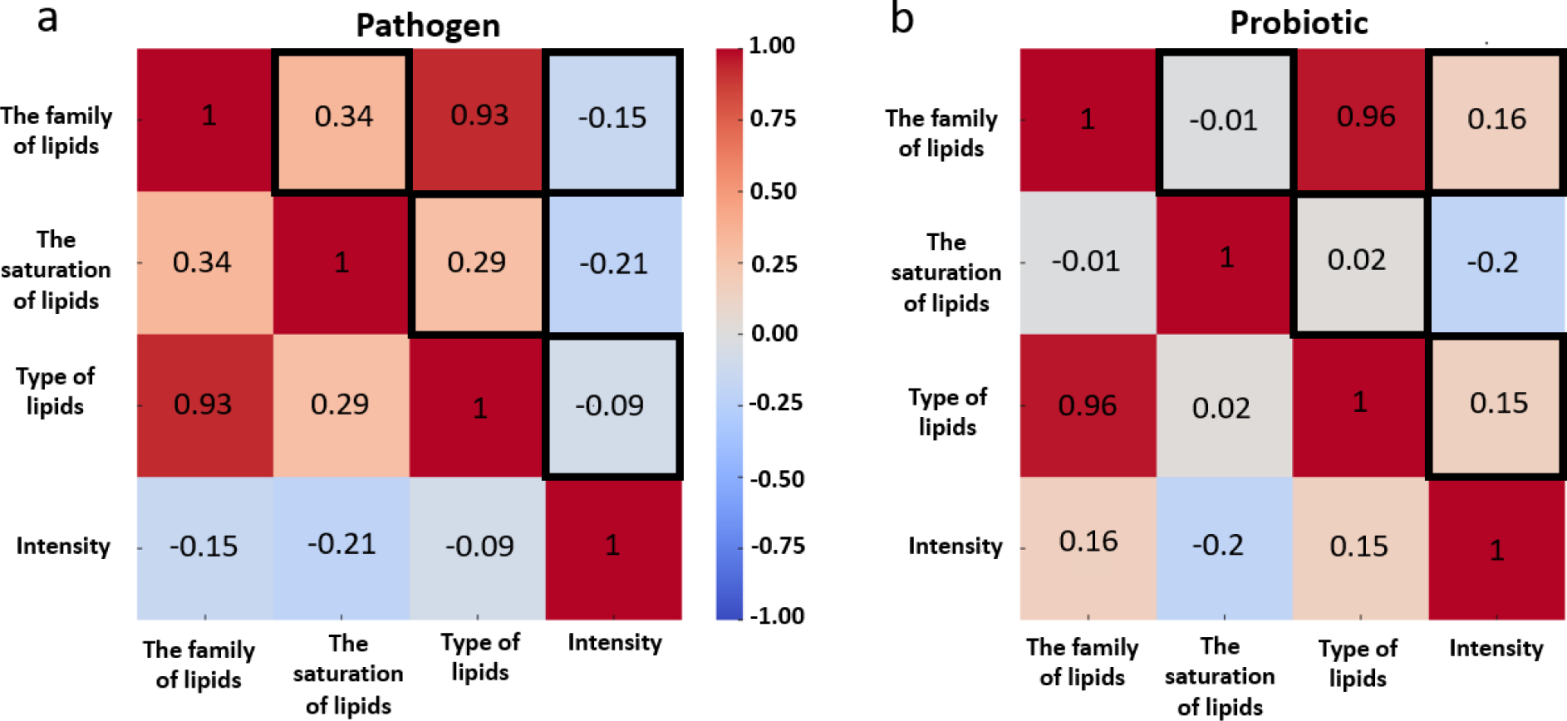
Pearson’s correlation matrices for the analyzed features in pathogenic (a) and probiotic (b) bacteria. The black frame marks statistically significant differences in correlation patterns between the two groups.

In order to determine whether a significant correlation exists between pathogen and probiotic bacteria, the distribution of bacterial types across variables was analyzed. The Chi-Square Test for categorical variables indicates a statistically significant association between the pathogen and probiotic bacteria, highlighting significant differences in their distribution (Table S2). Additionally, the Mann–Whitney U test for continuous variables demonstrated a significant difference (p-value = 0.00031) in intensity between pathogenic and probiotic bacteria. Distinct numbers and types of lipids were identified between pathogenic and probiotic bacteria. For example, LPE and PC were present only in pathogen samples, while a statistically significant greater number of PE and PG lipids were observed in pathogenic bacteria compared to probiotic bacteria. The intensity of lipid signals also varied, with higher intensities for LPG and PG found in pathogenic bacteria than in probiotic strains. These findings highlight the metabolic flexibility of pathogens, which necessitate a diverse array of lipids to adapt to various stresses, including oxidative stress and antimicrobial pressures. Conversely, probiotic bacteria, residing in stable and nutrient-rich environments like the gut, exhibit a more streamlined lipid composition that supports gut health and nutrient absorption. Different correlation patterns were observed for probiotic bacteria compared to pathogenic bacteria (Figure 7). Significant differences were found between the family of lipids with lipid intensity and type of lipid saturation, the saturation of lipids with type of lipids, and the type of lipids with intensity for the two sets of correlation coefficients. A positive correlation was demonstrated between the family of lipids and their saturation in pathogenic bacteria, while a negative correlation was observed in probiotic bacteria. A similar correlation pattern was noted for lipid saturation and lipid type, underscoring significant differences in lipid-related variables between the two bacterial groups. The differences in lipid saturation levels further emphasize the distinct roles of these bacteria. For instance, polyunsaturated fatty acids were dominant in pathogenic strains, whereas monounsaturated fatty acids were more prevalent in probiotics, suggesting differing metabolic requirements and environmental adaptations. This divergence in lipid profiles not only reflects the physiological needs of each bacterial group but also their strategies for survival and interaction with the host, ultimately influencing their pathogenicity or probiotic efficacy. In summary, the integration of statistical analyses with lipid profiling provides a comprehensive understanding of the distinct metabolic pathways and functional properties that differentiate pathogenic and probiotic bacteria.

### Exploring Lipid Composition Variations across Isolation Sources in Bacteria

The composition of bacterial membranes, including their lipid profiles, can vary depending on the matrix from which the bacteria are isolated. This variation may reflect adaptations to different environmental conditions and could influence bacterial physiology and pathogenicity. In this study, we investigated the impact of isolation matrix on the lipid profiles and membrane lipid content of three selected bacterial strains isolated from both clinical and environmental samples.

**Figure 8:**
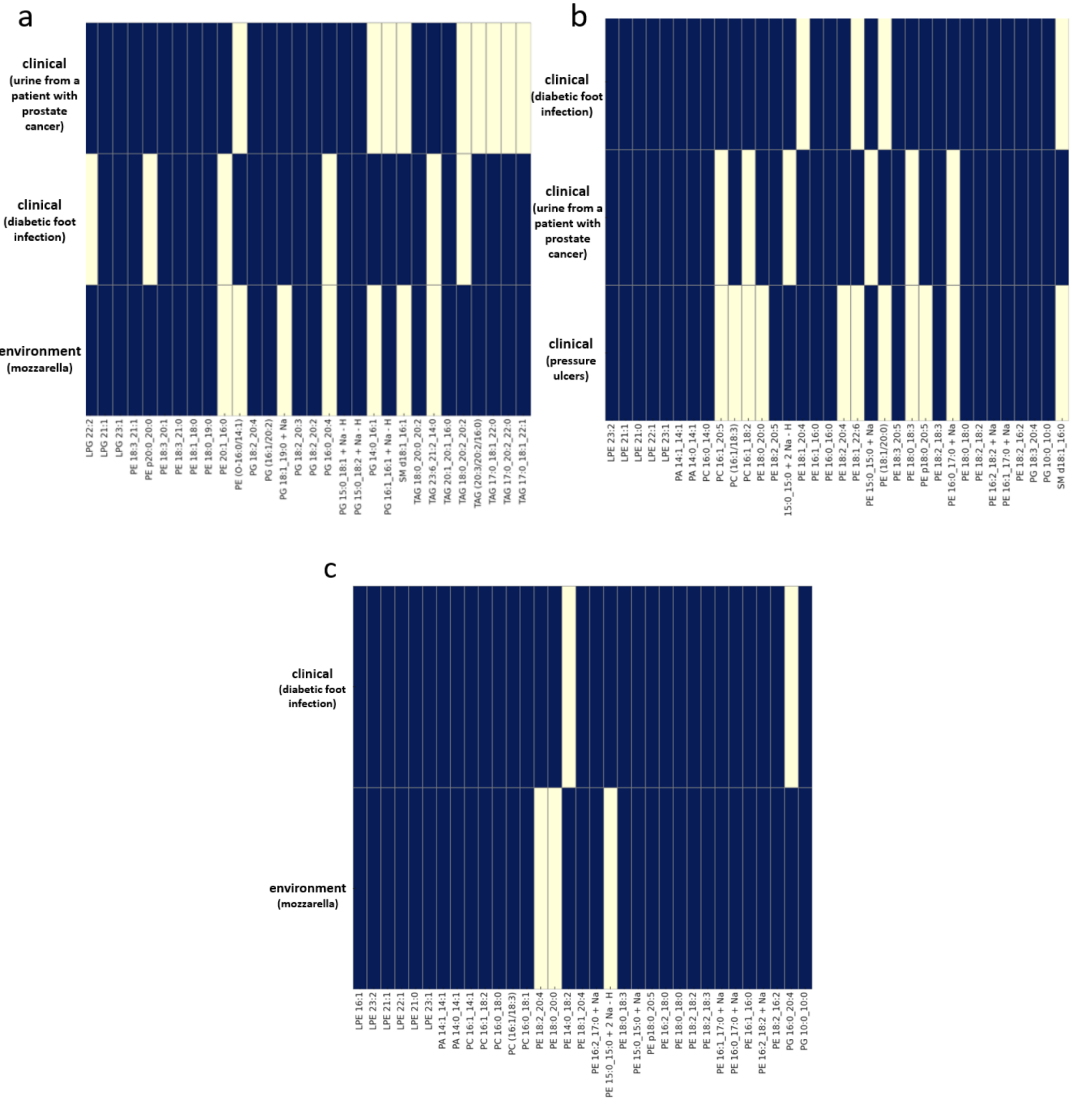
Heatmap of the presence of different lipid types across various types of matrices for *Staphylococcus epidermidis* (a), *Escherichia coli* (b) and *Citrobacter freundii* (c). In this visualization, a darker color indicates the presence of a specific lipid type, while yellow signifies its absence.

Individual species isolated from various clinical and environmental matrices demonstrated unique lipid profiles (Figure 8). Lipids such as LPG 21:1 and PE 18:3_20:1 are examples found in the lipid profile of *Staphylococcus epidermidis* regardless of the bacterial isolation source. PG 18:1_19:0 + Na is present in clinical samples and absent in environmental samples. Conversely, lipids such as PE 20:1_16:0, PG 16:0_20:4, and TAG 23:6_21:2_14:0, are matrix-dependent lipids that occurred only in specific clinical samples (urine) (Figure 8a). *Escherichia coli* isolated from different matrices demonstrated a higher number of matrices-independent lipids. Lipids such as LPE 23:2, LPE 21:1, LPE 21:0, PA 14:1_14:1, PA 14:0_14:1, PE 16:1_16:0, and PG 18:3_20:4 were identified across all matrices. In contrast, PC 16:1_18:3, PE 18:2_20:4, and PE 18:0_20:5 were found in clinical samples from diabetic foot infections and urine from oncology patients, but were absent in samples from pressure ulcers. Additionally, other lipids, such as PC 16:1_20:5, PC 16:1_18:2, and PE 18:0_18:3, were exclusively identified in specific samples (pressure ulcers) (Figure 8b). In *Citrobacter freundii*, a high number of lipids were present in both clinical and environmental matrices, although some were specific to certain matrices. PE 18:2_20:4, PE 18:0_20:0, and PE 15:0_15:0 + 2 Na – H were specifically identified in clinical samples from diabetic foot infections. Conversely, PE 14:0_18:2 and PG 16:0_20:4 were uniquely identified in mozzarella samples (Figure 8c).

Our investigation into the lipid composition of bacterial membranes indicates considerable variability contingent upon the source matrix of bacterial isolation. Our results substantiate that environmental elements, including temperature fluctuations, oxidative stress, substrate availability, and chemical exposure, exert a substantial influence on the lipid diversity within bacterial membranes. Additionally, factors such as pH levels, osmotic pressure, and the presence of antimicrobial agents may also contribute to variations in membrane lipid content.^38,39^ We observed that bacteria isolated from different matrices exhibit distinct lipid profiles, suggesting that bacterial membrane adaptation to changing environmental conditions is a dynamic and complex process. Specifically, temperature appears to be a key factor influencing the lipid composition of membranes, with variations in temperature leading to changes in the proportions of different lipid classes and in the structure of the acyl chain.^40^ Furthermore, the presence of oxidative stress, generated by free radicals, has been observed to induce modifications in bacterial membrane lipids, potentially altering membrane stability and functionality.^41^ Membrane fluidity is essential for cell viability, and bacterial cells can modify this property in response to external conditions by changing the fatty acid composition, chain length, and phospholipid content of their membranes. To counteract the fluidizing effects of hydrophobic compounds, bacteria often adjust their membrane composition to decrease fluidity and maintain a balance between bilayer and nonbilayer forming phospholipids. These modifications are crucial for surviving high concentrations of toxic contaminants. The discovered that anaerobic bacteria are approximately three times more sensitive to various organic compounds than aerobic bacteria, probably due to their slower growth rates.^42^ Emerging pollutants like tetrabromobisphenol A (TBBPA) alter bacterial lipid profiles and membrane permeability, impacting bacterial physiology and resistance. TBBPA disrupts membrane integrity by changing lipid composition and fluidity. This affects membrane permeability and cellular processes, hindering bacterial growth.^43^ It is noteworthy that the carbon source available to bacteria, originating from the growth medium on which they were cultivated, can significantly affect the composition of membrane lipids. This implies that carbohydrate metabolism plays a pivotal role in lipid biosynthesis, whereby the utilization of specific carbon sources may lead to differential regulation of lipid synthesis pathways and subsequent variations in membrane lipid content.^44^ Additionally, it is essential to consider that in the case of lipids extracted from bacteria derived from individuals with diabetic foot infections, who exhibit disrupted metabolism due to elevated blood glucose levels, this metabolic imbalance may also influence the lipid profile of these bacteria. Therefore, alongside environmental factors and carbon source availability, metabolic dysregulation in host organisms can further contribute to alterations in bacterial membrane lipid composition. First, it regulates nutrient availability in the host environment, affecting bacterial lipid synthesis pathways and membrane lipid composition. Fluctuations in glucose, fatty acids, and amino acids can directly modulate bacterial lipid metabolism. Second, the host immune response, especially during infection or inflammation, induces metabolic changes that influence bacterial lipid profiles. Immune cells release cytokines and reactive oxygen species, altering bacterial metabolism to adapt to the host environment.^45^ Hormonal regulation also shapes bacterial lipid metabolism, with hormones like insulin and cortisol influencing nutrient availability and potentially changing lipid profiles.^46^ Additionally, disease states like diabetes, obesity, or cancer can profoundly alter bacterial lipid profiles due to changes in nutrient availability, immune responses, and hormonal imbalances associated with these conditions. An interesting conclusion from our study is that the presence of chemicals, such as antibiotics, can alter the composition of bacterial membrane lipids. We find that exposure of bacteria to antibiotics may result in changes in lipid composition, with potential implications for antibiotic resistance and bacterial defense mechanisms. Furthermore, it is noteworthy that in the case of samples derived from individuals with diabetic foot infections, prostate cancer and pressure ulcers, antibiotics were administered to these individuals. This likely contributed to the distinct lipid composition observed in the bacterial membranes of these samples. Lee et al. work describes the impact of the use of antibiotics on the qualitative composition of lipids and fatty acids in bacteria. In daptomycin-resistant *S. aureus*, reduced odd-numbered carbon chain lipids and a shift toward longer acyl-chains were observed. Increased acyl-chain length correlated with reduced daptomycin-induced pore formation, suggesting resistance is driven by changes in acyl-chain compositions and reduced PG levels rather than membrane charge.^47^

The conclusions of our research suggest that the lipid diversity of bacterial membranes is the result of interactions between multiple environmental factors that regulate lipid biosynthesis, modification, and degradation processes. Understanding these mechanisms may be crucial for the development of novel therapeutic or biotechnological strategies based on manipulation of bacterial membrane lipid composition. However, it is worth noting that despite advancements in lipidomic research, there are still technical challenges associated with lipid identification errors and quantitative determination of lipid abundance. Nevertheless, our study represents a significant step toward better understanding the complex mechanisms of bacterial adaptation to changing environmental conditions.

## Conclusions

The conclusions drawn from our study underscore the significance of intricate interactions between environmental factors and the lipid composition of bacterial membranes. Noticeable differences in lipidomic profiles between Gram-positive and Gram-negative bacteria, as well as between pathogenic and probiotic strains, suggest the existence of complex adaptive mechanisms shaping the lipid composition of cell membranes. Additionally, the diverse lipid compositions exhibited by bacteria isolated from various sources, such as diabetic foot infections and clinical samples from pressure ulcers, emphasize the crucial role of the environment in regulating bacterial lipid biosynthesis.

It is important to acknowledge that the omission of outer membrane lipids, which are known to play a significant role in the pathogenesis of Gram-negative bacteria, limits our understanding of the complete lipidomic landscape. While our focus was on lipid classes that could be reliably analyzed using the chosen methodologies, future studies should indeed incorporate the analysis of OM lipids to provide a more comprehensive view of lipid profiles in both pathogenic and probiotic bacteria. Understanding these complex interactions could help elucidate the role these microorganisms play in the context of pathogenicity.

These insights hold promise for the development of novel therapeutic and biotechnological strategies aimed at manipulating bacterial lipid composition. Understanding the underlying adaptive mechanisms could offer new avenues for combating antibiotic resistance and improving public health outcomes. Nevertheless, it is crucial to recognize the inherent technical challenges associated with lipid identification errors and quantification of lipid abundance. Further research endeavors are warranted to fully elucidate the complex interrelationships governing bacterial lipid biology and to harness its potential for advancing human health and well-being.

## Supporting Information Available

All identified combinations of fatty acids in lipid molecules species are present in Table S1. In Table S2 present Chi-square test results with corresponding p-value for lipid-related categorical variables (PDF)
