# Supplementary material for: Environmental Influence on Bacterial Lipid Composition: Insights from Pathogenic and Probiotic Strains
Source: ACS Omega. 2024 Aug 29;9(36):37789–801. doi: 10.1021/acsomega.4c03778 (PMC11391446; doi:10.1021/acsomega.4c03778)
Supplement: Supplementary file 1 — ao4c03778_si_001.pdf [file ao4c03778_si_001.pdf]

Environmental Influence on Bacterial Lipid Composition: Insights from Pathogenic and Probiotic Strains

Justyna Walczak-Skierska[1*], Agnieszka Ludwiczak[1,2], Ewelina Sibińska[1], Paweł Pomastowski[1]

[1]*Centre for Modern Interdisciplinary Technologies, Nicolaus Copernicus University in Toruń, Wileńska 4 Str., 87-100 Toruń, Poland*

[2]*Faculty of Biological and Veterinary Sciences, Nicolaus Copernicus University in Toruń, Lwowska 1 Str., 87-100 Toruń, Poland*

**corresponding author: walczak-skierska@umk.pl*

Table S1. Identified combinations of fatty acids in lipid molecules species for all bacteria.

LP	m/z	LIPID	Citrobacter freundii G-		Lactobacillus plantarum G+		Enterococcus faecalis G+		Pseudomonas aeruginosa G-		Staphylococcus aures G+		Staphylococcus epidermidis G+			Escherichia coli G-			Proteus mirabilis G-		Klebsiella pneumoniae G-		Lactococcus lactis G+		
			M*	DFI 35	C*	B*	DFI 44	U7.5	DFI 10	U10.68	DFI 35	U10.11	DFI 38	U7.03	M*	PU	DFI 34	U9.62	DFI 39	U11.20	DFI 12	U11.40	milk	B*	M*
1	428	LPG 12:0			+	+																			
2	436	LPA 18:1															+								
3	454	LPE 16:1	+	+																					
4	493	LPC 16:1									+														
5	521	LPE 21:1	+	+												+	+	+	+	+		+			
6	522	LPC 18:1			+																				
7	523	LPE 21:0	+	+							+	+				+	+	+	+	+	+	+			
8	535	LPE 22:1	+	+												+	+	+	+	+					
9	544	LPC 20:4				+																			
10	547	LPE 23:2	+	+												+	+	+	+			+			
11	549	LPE 23:1	+	+				+								+	+	+	+	+	+	+			
12	551	LPG 21:1									+	+	+	+	+								+		
13	565	LPG 22:2									+	+		+	+										
14	577	PG 10:0_10:0	+	+	+	+	+	+	+	+						+	+	+	+	+	+	+	+		+
15	579	LPG 23:1									+	+	+	+	+				+						
16	589	PA 14:1_14:1	+	+			+	+				+				+	+	+	+		+	+			
17	591	PA 14:0_14:1	+	+		+	+	+								+	+	+	+		+	+	+	+	+
18	592	LPC 23:1																		+					
19	610	PG 12:0_12:0			+																				+
20	647	PE O-16:0_14:1											+									+			
21	655	PE 12:0_18:4					+	+																	
22	686	PE 15:0_15:0 + Na	+	+												+	+		+	+	+	+			
23	688	PE 14:0_18:2	+																						
24	690	PE 16:1_16:0	+	+												+	+	+				+			
25	691	PE 16:0_16:0														+	+	+		+	+				
26	693	PG 14:0_16:1											+									+			
27	701	SM d18:1_16:1			+	+	+			+			+												+

28	702	PC 16:1_14:1	+	+																	+	+			
29	703	SM d18:1_16:0																+				+			
30	705	PC 16:0_14:0							+	+						+	+	+	+	+		+			
31	708	PE 15:0_15:0 + 2 Na – H		+												+	+			+					
32	712	PE 18:2_16:2	+	+												+	+	+	+	+	+	+			
33	716	PE 16:2_18:0	+	+																					
34	717	PG 15:0_15:0 + Na									+	+													
35	720	PE 16:0_18:0																					+	+	
36	724	PE 16:2_17:0 + Na	+	+																					
37	725	PG 14:0_15:0 + 2Na									+													+	
38	726	PE 16:1_17:0 + Na	+	+												+	+	+	+	+	+	+			
39	728	PE 16:0_17:0 + Na	+	+													+		+	+	+	+			
40	732	PC 16:0_16:1								+															
41	733	PG 16:1_17:1									+	+													
42	734	PC 16:0_16:0							+	+															
43	734	PE 16:2_18:2 + Na	+	+												+	+	+	+	+	+	+			
44	738	PC 16:1_15:1 + Na							+	+															
45	738	PE 18:2_18:3	+	+												+	+	+			+	+			
46	739	PG 16:1_16:1 + Na – H									+	+	+		+								+		
47	740	PC 16:0_15:1 + Na							+	+															
48	740	PE 18:2_18:2	+	+												+	+	+	+	+	+	+			
49	742	PE 18:0_18:3	+	+													+		+	+	+	+			
50	745	PE 18:1_18:0									+	+	+	+	+										
51	747	PE 20:1_16:0									+			+											
52	748	PE 18:0_18:0	+	+												+	+	+	+	+	+	+			
53	750	PE p18:0_20:5	+	+													+	+	+	+	+	+			
54	753	PG 15:0_18:2 + Na – H									+	+	+	+	+										
55	754	PC 16:1_18:3	+	+						+							+	+	+		+	+			
56	755	PG 15:0_18:1 + Na – H									+	+	+	+	+										
57	756	PC 16:1_18:2	+	+						+							+		+		+	+			
58	758	PC 16:0_18:2							+	+															
59	760	PE 18:2_20:5														+	+	+							

60	760	PC 16:0_18:1	+	+					+	+												+			
61	761	PE 18:0_19:0									+	+	+	+	+										
62	762	PE 18:3_20:5														+	+	+	+	+					
63	762	PC 16:0_18:0	+	+					+	+											+	+			
64	764	PE 18:2_20:4		+													+	+		+	+	+			
65	766	PE 18:1_20:4	+	+												+		+			+	+			
66	767	PE 18:3_20:1									+	+	+	+	+										
67	769	PG 16:0_20:4	+							+	+			+											
68	773	PG 16:1_20:2									+	+	+	+	+										
69	774	PE 18:1_20:0																+							
70	776	PE 18:0_20:0		+						+							+	+		+	+	+			
71	778	PE 17:0_20:3 + Na																		+					
72	778	PC 16:1_20:5							+	+							+				+	+			
73	779	PG 16:0_20:0																						+	
74	780	PC 18:2_18:3								+										+	+	+			
75	781	PE 18:3_21:1									+	+	+	+	+										
76	782	PC 18:1_18:3							+	+											+	+			
77	783	PE 18:3_21:0						+					+	+	+										
78	783	PG p18:0_18:1 + Na									+	+													
79	788	PE 18:1_22:6								+								+							
80	789	PE p20:0_20:0												+	+										
81	793	PG 18:3_20:4			+	+				+						+	+	+		+			+	+	+
82	794	PE 20:1_20:4																			+	+			
83	795	PG 18:2_20:4									+	+	+	+	+										
84	797	PG 18:2_20:3									+	+	+	+	+										
85	798	PE 20:1_18:2								+															
86	799	PG 18:2_20:2									+	+	+	+	+										
87	801	PE 20:1_20:1						+															+		
88	802	PC 18:3_20:5								+															
89	807	PG 20:0_18:0					+	+		+													+	+	+
90	813	PG 18:1_19:0 + Na									+		+	+											
91	823	PG 18:2_20:1 + Na						+			+	+											+	+	+
92	828	PC 20:2_19:0								+															
93	830	PC 20:1_19:0								+															

94	846	PC 20:0_20:0									+	+													
95	852	PE 21:1_23:3									+	+													
96	856	PE 22:1_22:1								+															
97	874	TAG 15:0_17:0_21:1									+	+													
98	877	PG 22:0_21:0			+		+	+																	
99	884	TAG 20:5_20:5_15:0									+	+													
100	888	TAG 20:5_20:3_15:0									+	+													
101	889	TAG 18:0_18:0_18:1					+	+																	
102	891	TAG 18:0_18:0_18:0					+	+																	
103	896	TAG 22:6_20:4_14:1									+	+													
104	900	TAG 16:0_19:1_20:1									+														
105	902	TAG 15:0_21:2_20:6									+	+													
106	909	TAG 20:3_20:2_16:0											+		+										+
107	912	TAG 14:0_20:4_23:6									+	+													
108	913	TAG 20:1_20:2_16:0			+	+	+	+															+	+	+
109	915	TAG 20:1_20:1_16:0			+	+		+					+	+	+								+	+	+
110	916	TAG 14:0_20:2_23:6									+	+													
111	917	TAG 20:1_20:0_16:0					+	+																	
112	927	TAG 17:0_18:2_22:1				+	+	+															+	+	+
113	929	TAG 17:0_18:1_22:1			+	+	+	+					+		+										
114	930	TAG 23:6_21:2_14:0									+	+		+											
115	931	TAG 17:0_18:1_22:0											+		+								+	+	
116	932	TAG 23:6_21:0_14:1									+	+													
117	935	TAG 18:2_20:2_20:2					+	+																	+
118	939	TAG 18:0_20:2_20:2			+	+	+	+							+										+
119	941	TAG 18:0_20:1_20:2			+	+	+	+															+	+	+
120	942	PC 24:6_24:2							+	+															
121	943	TAG 18:0_20:0_20:2			+			+					+	+	+								+	+	
122	944	TAG 23:6_20:2_16:0							+		+	+													
123	953	TAG 17:0_20:3_22:1				+	+	+																+	+
124	954	TAG 17:1_18:2_24:0							+	+															
125	955	TAG 17:0_20:2_22:1			+	+	+	+															+	+	+
126	957	TAG 17:0_20:2_22:0			+	+	+	+					+		+										
127	958	TAG 23:6_20:2_17:0									+	+													

128	965	TAG 16:1_22:0_22:0																							+
129	967	TAG 16:1_22:0_22:1				+	+	+															+		+
130	969	TAG 16:1_22:0_22:2			+	+		+															+	+	+
131	971	TAG 16:0_22:0_22:2			+	+	+	+															+	+	+
132	997	TAG 20:0_20:2_22:1					+	+																	
133	1093	TAG 28:2_22:2_19:0									+														
134	1374	CLP 16:0_16:0_16:0_16:1	+																						
135	1396	CLP 16:1_17:1_16:1_17:0	+																						
136	1489	CLP 18:0_18:0_18:0_18:0																+		+					

M* - mozzarella; C* - cucumber; B* - beetroot

„+" means lipid molecular species identified

Table S2. Chi-square test results with corresponding p-value for lipid-related categorical variables.

Categorical variable	Chi-Square Value	p-value
Type of bacteria	108.97	1.65×10^{-25}
The family of lipids	122.44	1.04×10^{-22}
The saturation of lipids	9.58	0.0225
Type of lipids	294.71	1.26×10^{-14}
The type of saturation	262.01	6.86×10^{-17}
